# Chemical vs. Physical Methods to Improve Dermal Drug Delivery: A Case Study with Nanoemulsions and Iontophoresis

**DOI:** 10.3390/pharmaceutics14061144

**Published:** 2022-05-27

**Authors:** Ines Nikolić, Mitar Simić, Ivana Pantelić, Goran Stojanović, Jelena Antić Stanković, Bojan Marković, Snežana Savić

**Affiliations:** 1 Department of Pharmaceutical Technology and Cosmetology, University of Belgrade—Faculty of Pharmacy, 11000 Belgrade, Serbia; ines.nikolic@pharmacy.bg.ac.rs (I.N.); ivana.pantelic@pharmacy.bg.ac.rs (I.P.); snezana.savic@pharmacy.bg.ac.rs (S.S.); 2 Department of Electronics, University of Novi Sad—Faculty of Technical Sciences, 21000 Novi Sad, Serbia; mitar.simic@uns.ac.rs (M.S.); sgoran@uns.ac.rs (G.S.); 3 Department of Microbiology and Immunology, University of Belgrade—Faculty of Pharmacy, 11000 Belgrade, Serbia; jelena.stankovic@pharmacy.bg.ac.rs; 4 Department of Pharmaceutical Chemistry, University of Belgrade—Faculty of Pharmacy, 11000 Belgrade, Serbia

**Keywords:** dermal drug delivery, iontophoresis, penetration enhancers, nanoemulsions, curcumin

## Abstract

So far, various approaches have been proposed to improve dermal drug delivery. The use of chemical penetration enhancers has a long history of application, while methods based on the electrical current (such as iontophoresis) stand out as promising “active” techniques. Aiming to evaluate the contribution of different approaches to dermal delivery, in this work curcumin-loaded nanoemulsions with and without monoterpenes (eucalyptol or pinene) as chemical penetration enhancers, and a custom-made adhesive dermal delivery system based on iontophoresis were designed and assessed. In an in vivo study applying skin bioengineering techniques, their safety profile was proven. Three examined iontophoresis protocols, with total skin exposure time of 15 min (continuous flow for 15 min (15-0); 3 min of continuous flow and 2 min pause (3-2; 5 cycles) and 5 min of continuous flow and 1 min pause (5-1; 3 cycles) were equally efficient in terms of the total amount of curcumin that penetrated through the superficial skin layers (in vivo tape stripping) (Q_3-2_ = 7.04 ± 3.21 μg/cm^2^; Q_5-1_ = 6.66 ± 2.11 μg/cm^2^; Q_15-0_ = 6.96 ± 3.21 μg/cm^2^), significantly more efficient compared to the referent nanoemulsion and monoterpene-containing nanoemulsions. Further improvement of an efficient mobile adhesive system for iontophoresis would be a practical contribution in the field of dermal drug application.

## 1. Introduction

The skin, as the largest organ of the human body, has numerous and very important features. Not only does it represent a powerful protective barrier, but it provides communication with the external environment, participates in thermoregulation, possesses hormonal activity, and plays a notable role in achieving an adequate immune response [1].

The application of medicinal compounds on the skin has been known since ancient times [2]. In modern pharmacotherapy, this type of treatment is very complex and abounds in preparations for various indications. However, numerous scientific findings have enabled us to use the skin not only to achieve local treatment, but also for some significant effects in the deeper layers of the skin, and even for transdermal drug delivery. Precisely, in the context of contemporaneous tendencies in the development of pharmaceutical dosage forms, which aim to provide patients with a simple, least invasive and, of course, effective therapy, the skin represents an attractive application site, providing possibilities for both local and systemic effects. Availability of the active ingredient at the target site of action is increased, while systemic side effects are reduced. The administration of the drug itself is simple, which leads to better patient adherence and more successful treatment outcomes. However, regardless of whether it is necessary to achieve effects on the surface, in its deeper layers (in the viable epidermis or dermis), or the goal is to deliver the active into the systemic circulation, administration on the skin is a certain challenge due to its heterogeneous structure [1,3].

The main hurdle for active ingredients to reach the anticipated site of action upon dermal application is represented in the dense, keratinized superficial skin layer—*stratum corneum* [4]. In the literature, a pictorial description of “brick and mortar” is often used when presenting characteristics of this layer, suggesting its barrier properties due to interconnected corneocytes (“bricks”) immersed into the lipid matrix (“mortar”). In addition to this, completely opposite surrounding is found in dermis, which abounds in glycosaminoglycans and structural proteins, containing blood and lymph vessels, sweat and sebaceous glands, and hair follicles. Such a complex structure strongly affects delivery of actives [1].

Various approaches have been proposed to improve dermal availability, and all developed methods can be classified into passive (implying that the active ingredient passes through the *stratum corneum* by passive diffusion after “chemical” reduction of its permeability) and active ones (providing an external driving force that “pulls” the molecules of the active ingredient through the epidermis) [4,5,6].

Among passive methods to improve dermal availability, the use of chemical penetration enhancers is probably the most common approach, with a long history of application [7]. So far, it has been shown that more than 350 molecules can increase skin permeability through several mechanisms [5]. Among a few classes of recognized chemical penetration enhancers (including surfactants, short-chain alcohols and glycols, fatty acids, and their esters), terpene compounds stand out, being established as effective penetration enhancers with lower skin irritation compared to conventional, synthetic ones [8,9]. However, available literature sources have not provided significant evidence-based pieces of information regarding terpene-governed penetration enhancement effect in humans [10]. Considering previous research focused on co-stabilization effects of eucalyptol and pinene in nanoemulsions [11], the authors now aimed to evaluate their effect in terms of skin delivery.

Nevertheless, passive methods are not able to provide an adequate level of penetration for molecules of higher molecular weight, or for molecules with more polar structure. In this domain, active methods could give significantly better outcomes [4]. Perhaps the most commonly used ways to improve dermal delivery among active methods are iontophoresis and electroporation—methods based on the application of electrical energy [12,13]. However, despite their effectiveness, not much has been done in terms of their realistic application. Moreover, skin tolerability aspect is frequently neglected.

Iontophoresis represents topical application of low-density electrical current (less than 0.5 mA/cm^2^) during several minutes or several hours (in continuous or discontinuous mode), in combination with a drug formulation, which facilitates transport through the skin and other tissues/biological membranes. Although it is predominantly used for the delivery of small (molecular weights up to several kDa) polar/charged molecules, the great advantage of iontophoresis is reflected in the ability to provide (trans)dermal delivery of peptides and oligonucleotides, which opens new fields in the drug delivery of biologics [12,14,15].

In this research, curcumin was used as a model active ingredient. This compound has been known for its numerous beneficial properties, especially antioxidant, anti-inflammatory, antiproliferative and wound-healing. However, its physicochemical characteristics remain a challenge for its more realistic application. Therefore, the encapsulation of curcumin in various nano-enabled delivery systems has been proposed to maximize its potential, such as nanoemulsions [16], microemulsions [17], liposomes [18] or hybrid vesicles [19].

Relying on previously gained good experience with nanoemulsions as drug delivery systems [11,20,21], the authors aimed to position their effect in relation to another approach for dermal delivery improvement—iontophoresis. In this work, curcumin-loaded nanoemulsions with and without monoterpenes as chemical penetration enhancers, as well as a custom-made adhesive dermal delivery system based on iontophoresis were designed and assessed in terms of their safety profiles and ability to deliver curcumin into/through the skin. After thorough analysis, comparison of the two approaches was performed, with the ultimate idea to provide an efficient and acceptable system for improved dermal drug delivery, providing some new insights into the effects of iontophoresis.

## 2. Materials and Methods

### 2.1. Materials

The following components were used to prepare the blank and curcumin-loaded samples (nanoemulsions): medium chain triglycerides (MCT)—Mygliol 812 (Fagron, Rotterdam, The Netherlands), polysorbate 80 (Across Organics, Geel, Belgium), soybean lecithin with 70% of phosphatidylcholine—Lipoid S 75 (Lipoid, Ludwigshafen, Germany), 1,8-cineole (eucalyptol; Sigma Aldrich, St. Louis, MO, USA), pinene (Sigma Aldrich, St. Louis, MO, USA), highly purified water (GenPure, TKA Wasseranfbereitungssysteme GmbH, Niederelbert, Germany). Curcumin (Sigma Aldrich, St. Louis, MO, USA) was used as a model active ingredient. All other substances and reagents used in the experimental work were of pharmacopoeial (Ph. Eur.) or HPLC quality, and were used without further purification.

### 2.2. Methods

#### 2.2.1. Nanoemulsion Preparation

Nanoemulsions were prepared via a spontaneous emulsification procedure. Briefly, (for 20 g of the nanoemulsion formulation), lecithin (0.2 g) was dissolved in the oil phase (2 g; MCT or its combination with eucalyptol or pinene—50:50), and then polysorbate 80 (1.8 g) was added. The surfactant-to-oil ratio (SOR) was 1. After mixing on a magnetic stirrer, the oil-surfactant blend (4 g) was slowly added dropwise, during 5 min, to highly purified water (16 g), under constant magnetic stirring at 1000 rpm. After the complete amount of the oil-surfactant blend was added, mixing was continued for another 60 min. The prepared nanoemulsions were transferred to the glass bottles, hermetically sealed, and stored protected from light.

In the case of curcumin-loaded nanoemulsions, the preparation process was the same, only the defined amount of the active compound (0.06 g of curcumin for 20 g of the formulation) was first dissolved in the oil-surfactant blend, which was later added to the aqueous phase, in the manner already described. The final concentration of curcumin in the nanoemulsions was 3 mg/mL.

Initial characterisation of the blank and curcumin-loaded nanoemulsions was performed 24 h after preparation. Results of the long-term stability study of the designed formulations is provided in the Supplementary Materials section.

#### 2.2.2. Droplet Size and Size Distribution Analysis

For droplet size analysis, batch mode DLS technique was carried out, according to the standard operative procedure NCL-PCC-1 [22], applying Zetasizer Nano ZS90 (Malvern Instruments Ltd., Malvern, UK). Prior to measurements, all samples were diluted with ultrapure water (1:100 *v*/*v*) and transferred to a disposable polystyrene cuvette. The measurements were performed at 25 °C, using He-Le laser with wavelength of 633 nm, at a scattering detection angle of 90°. Equilibration time was set to 5 min, number of measurements per analysis was 10, and each measurement was conducted in 12 runs lasting 10 s each. Malvern Dispersion Technology Software—DTS (Nano), version 5.00 was used for data analysis. As relevant parameters, intensity-based droplet diameter (Z-ave) and polydispersity index (PDI) were considered, with simultaneous assessment of the graphical representation of the droplet size distribution.

#### 2.2.3. Determination of the Surface Charge of the Nanodroplets

The zeta potential, as an indicator of droplet surface charge, was determined with a Zetasizer Nano ZS90 (Malvern Instruments Ltd., Malvern, UK), measuring the electrophoretic mobility of droplets in an electric field. To prevent fluctuations in the electrical conductivity of water, prior to the measurement, the samples were diluted (1:100 *v*/*v*) with highly purified water containing NaCl, so that the conductivity was adjusted to 50 μS/cm. After dilution, samples were immediately transferred to the measuring cell with two electrodes.

#### 2.2.4. pH Measurements

The pH value of the formulation is important not only regarding the route of application, but also for maintaining the chemical stability and efficiency of the active molecules that are prone to chemical degradation under certain pH conditions (such as curcumin, which undergoes oxidative degradation in alkaline environment). Measurements were performed by direct immersion of the electrode of the pH meter HI9321 (Hanna Instruments Inc., Ann Arbor, MI, USA) in nanoemulsion samples, at 25 ± 2 °C.

#### 2.2.5. In Vitro Safety Assessment of the Nanoemulsions—Cytotoxicity Assay

Aiming to assess the safety profile of the formulations, in vitro cytotoxicity tests were performed on normal human keratinocytes (HaCaT), as the dominant structural cells of the epidermis. For this purpose, the MTT test was conducted—one of the commonly used tests that provide insight into the metabolic activity and cell viability. The MTT is a water-soluble yellow colour, and after reduction by enzymes in metabolically active cells, it turns into a blue-violet formazan, which can be determined colorimetrically. The intensity of the blue-violet colour corresponds to the metabolic activity of the cells, which can be related to their viability [23,24].

##### Cell Line

The cell line was obtained from American Type Culture Collection (Manassas, VA, USA). The cells were maintained in complete nutrient medium RPMI-1640 at 37 °C, in a humidified atmosphere with 5% CO_2_. The RPMI 1640 (Sigma–Aldrich, St. Louis, MO, USA) was used as the nutrient medium, supplemented with L-glutamine (3 mM), streptomycin (100 mg/mL), penicillin (100 IU/mL), and foetal bovine serum (10%; heat-inactivated at 56 °C for inactivation of cholinesterase, system complement and HEPES (25 mM)). The pH was adjusted to 7.2 by bicarbonate solution.

##### Determination of the Cell Survival

Cell survival was determined by the MTT (3-(4,5-dimethylthiazol-2-yl)-2,5-diphenyl tetrazolium bromide) test, 48 h after the addition of the test samples. The MTT (Sigma-Aldrich, St. Louis, MO, USA) was dissolved in phosphate buffered saline (PBS, pH 7.2) in the concentration of 5 mg/mL and filtered through Millipore filters (0.22 μm) prior to use. In the test, 10 μL of the prepared MTT solution was added to each well. The samples were incubated for additional 4 h at 37 °C, in 5% CO_2_ and humidified atmosphere. Then, 10% sodium dodecylsulfate solution (SDS, Sigma-Aldrich, St. Louis, MO, USA) was prepared, and 100 μL of the solution was added to each of the wells. The next day, the absorbance of the cell medium from each well was measured at 570 nm. Measurements were performed using Multiskan^™^ FC Microplate Photometer (Thermo Scientific, Waltham, MA, USA). For cell survival calculations (%), the following equation was used:

(1) $$Cell\;survival\;\left(\%\right)=\frac{A_{test\;sample}-A_{blank\;sample}}{A_{control}-A_{blank\;sample}}\cdot 100$$

As the relevant parameter, IC_50_ was determined—the concentration of the sample which decreases survival of the treated cells by 50%.

#### 2.2.6. In Vivo Assessment of the Irritation Potential of the Blank Nanoemulsions

The relatively high concentration of surfactants in the nanoemulsions, and especially inclusion of terpenes as chemical penetration enhancers, may be responsible for potential skin irritation at the application site. For this reason, an in vivo study applying skin bioengineering techniques was conducted to assess the irritating potential (safety) of the selected formulations.

The study involved eight volunteers (aged from 23 to 29 years), who were informed about the study and gave their written consent to participate. Subjects did not use cosmetic products on the volar sides of the forearms 24 h before the experiment, while they did not consume caffeinated beverages 3 h before the study and during the study itself.

On the day of the test, each subject spent 30 min in the laboratory before the start of the experiment, in order to acclimatise. Afterwards, the test sites on the volar sides of the forearms (surface area for each test place: 4 cm^2^) were defined for the following samples:

- nanoemulsion without chemical penetration enhancers (F1);
- nanoemulsion with pinene as a chemical penetration enhancer (F1_PIN);
- nanoemulsion with eucalyptol as a chemical penetration enhancer (F1_EUC).

One place was marked as the control place, and it was not treated.

The values of the following parameters were measured at the test sites and at the control site:

- trans-epidermal water loss (TEWL);
- erythema index (EI);
- degree of hydration of the *stratum corneum* (stratum corneum hydration—SCH).

After the initial measurements, 250 μL of each formulation were applied to the marked places, and the same parameters were determined after 15, 60 and 120 min, accompanied by visual inspection of the treated skin.

The TEWL is a traditionally used indicator of skin barrier integrity. The measurement is based on the detection of water evaporation from skin surface (expressed in g/m^2^·h). An increase in TEWL indicates damage to the skin integrity (its superficial layers). This parameter was determined using a Tewameter^®^ 210 (Courage + Khazaka, Köln, Germany).

The EI represents a parameter that indicates the appearance of redness of the skin, which is determined spectrophotometrically, at the wavelength of haemoglobin. The Mexameter^®^ MX 18 (Courage + Khazaka, Köln, Germany) was used to determine this parameter.

The SCH is a parameter that can be determined by measuring the electrical conductivity of the skin. It was measured using a Cutometer^®^ MPA 580 (Courage + Khazaka, Köln, Germany).

All measurements were performed according to the relevant guidelines [25,26,27]. Parameter values at defined time points were compared with the initial ones (before the treatment), as well as with the nontreated control.

The study protocol was approved by the Ethics Committee of the Faculty of Pharmacy in Belgrade (decision No. 1579/2).

#### 2.2.7. In Vivo Penetration Study of Curcumin from Developed Curcumin-Loaded Nanoemulsions

Aiming to evaluate the penetration efficiency of curcumin from the developed formulations (formulation without chemical penetration enhancers and formulations with pinene and eucalyptol), an in vivo study was conducted which included four volunteers (aged 23 to 29 years). The penetration of curcumin through the superficial layers of the skin was determined by the tape stripping method, which was recently included by the European Medicines Agency in the Draft guideline on quality and equivalence of topical products [28].

The protocol for conducting this study was approved by the Ethics Committee of the Faculty of Pharmacy in Belgrade (decision No. 1579/2). Study participants were informed in detail about the examination protocol and gave their written consent to participate in the study. Subjects did not use cosmetic products on the volar sides of the forearms 24 h before the experiment, while 3 h before the study and during the study did not consume caffeinated beverages.

In order to acclimatise, before starting the experiment, each participant spent 30 min in the laboratory.

On the inner sides of the forearms, using a cardboard template, the test sites for the application of curcumin-loaded nanoemulsions were defined—three different formulations:

- formulation with pinene (F1_PIN_CU);
- formulation with eucalyptol (F1_EUC_CU);
- formulation without monoterpenes (F1_CU).

The initial TEWL values were measured, and then the formulations were carefully applied on defined test sites (250 μL of each formulation was applied to an area of 4 cm^2^). After 2 h, successive removal of 12 adhesive tapes was performed (D-Squame, Monaderm, Monaco; tape surface: 3.8 cm^2^). Each adhesive tape was individually placed on the treated site on the skin, and constant pressure of 140 g/cm^2^ was applied (using a spring-based device) for 10 s, which was followed by tape removal. In this way, 12 adhesive tapes were applied, and 12 “layers” of *stratum corneum* were removed from each test site. The mass of individual tapes was measured before and after application utilising an analytical balance Sartorius BP210D (Sartorius, Germany), to obtain data on the mass of removed corneocytes (gravimetric approach). The adhesive tapes were handled carefully, using tweezers, in order not to be contaminated by the researcher’s corneocytes. The TEWL measurement was repeated after 4th, 8th and 12th tape, to estimate the total *stratum corneum* thickness of the subjects.

After measuring the mass, each adhesive strip was carefully transferred to a cuvette containing 4 mL of 50% *v*/*v* of ethanol, which was used as a solvent for curcumin extraction. The cuvettes were then sonicated in a Sonorex RH102H ultrasonic bath (Bandelin, Berlin, Germany) for 15 min, and afterwards centrifuged for 5 min at 4000 rpm (Centrifuge MPW-56, MPW Instruments, Warsaw, Poland). A small amount of supernatant was transferred to glass vials and subjected to the LC-MS/MS analysis for curcumin concentration determination.

#### 2.2.8. Iontophoresis

Aiming to propose an adequate protocol for conducting iontophoresis as a physical method for improvement of the dermal availability of active ingredients, which would be suitable for application in the form of an adaptive adhesive system (patch), based on literature research, three different sets of conditions were proposed (1 with continuous current, and 2 with intermittent current), which were then subjected to an in vivo safety assay and then to the evaluation of the efficiency in the delivery of curcumin from the previously formulated nanoemulsion.

##### Irritation Potential Assessment of the Proposed Iontophoresis Protocols

After obtaining the permission of the Ethics Committee of the Faculty of Pharmacy in Belgrade (decision No. 1579/2), an in vivo study was conducted including eight volunteers (aged from 23 to 29) who were informed in detail about the study and gave their written consent to participate in the study. Subjects did not apply cosmetic products to the volar sides of the forearms 24 h before the study, while 3 h before and during the study they did not consume caffeinated beverages.

On the volar sides of the forearms, using a cardboard template, the positions for placing the electrodes linked to the adhesive patch (Skintact F-55) were marked. Electrodes were connected to the relay board supplied by 9 V power source. The relay board (and consequently the exposure time) was controlled with the microprocessor-based board. The control software for precise timing was implemented in the Arduino 1.8.11. development environment (Smart Projects, Pescara, Italy). The total exposure time to the current was 15 min, but in three different combinations:

- continuous flow for 15 min (15-0);
- 3 min of continuous flow and 2 min pause (3-2; 5 cycles);
- 5 min of continuous flow and 1 min pause (5-1; 3 cycles).

As the experiment involved testing under three different combinations of experimental conditions, seven places were defined: four on the left hand (for two protocols), two on the right (for the 3rd protocol) and another—control (nontreated) place on the right hand.

The parameters of interest (TEWL, EI, SCH) were measured initially and then in additional three time points: 15, 60 and 120 min after the iontophoresis. In addition to visual control, obtained values were compared with the initial ones as well as with the nontreated control, in the same way as described in the section ‘In vivo assessment of the irritation potential of nanoemulsions’.

##### In Vivo Penetration Study of Curcumin after the Application of Iontophoresis

After obtaining the permission of the Ethics Committee of the Faculty of Pharmacy in Belgrade (decision No. 1579/2), in vivo assessment of curcumin penetration through the superficial skin layers using iontophoresis as a physical method to improve dermal availability was performed by the tape stripping method. The study included four volunteers aged 23 to 29 years. All participants were informed in detail about the study and gave written consent for their participation.

In order to acclimatise, each subject spent 30 min in the laboratory before the start of the experiment. Applying cosmetic products to the volar sides of the forearms 24 h before the study, as well as consuming caffeinated beverages 3 h before and during the study were not possible.

On the inner sides of the forearms, using a cardboard template, places for positioning the electrodes were defined. As curcumin is in unionized form in the formulations, curcumin-loaded nanoemulsion (F1_CU) was applied to the site corresponding to the anode (“active electrode”). Initial TEWL values were measured at these sites, and then 250 μL of the formulation was applied to an area of 4 cm^2^. When the sample dried, the anode and cathode were attached, and the current flow was adjusted (for 15 min, in three different protocols). After 2 h of application of the formulation in combination with the iontophoresis, successive removal of 12 adhesive tapes, extraction and determination of curcumin content was performed as previously described in the section ‘In vivo penetration study of curcumin from the nanoemulsions’. In this way, all three iontophoresis protocols on each participant were successively examined.

It is worth mentioning that, when the electrodes are detached from the skin, due to the strong adhesive patch, a significant amount of the *stratum corneum* is removed (alongside with the active ingredient present in the upper parts of this layer), compromising the possibility to determine the exact amount of the active ingredient that penetrates the skin. Therefore, to facilitate the comparison of the two approaches (chemical penetration enhancers vs. iontophoresis), assessment of the curcumin penetration from the nanoemulsions without iontophoresis was performed again, but with slight protocol modification (used only for the purpose of comparison with iontophoretic protocol). Namely, in the case when iontophoresis was not used, the adhesive patch was applied to the treated place and removed after 15 min, while all other steps were as already described. Therefore, it was possible to remove approximately the same amount of *stratum corneum* as in the case of iontophoresis, thus enabling appropriate comparison.

#### 2.2.9. LC-MS/MS Analysis

Determination of curcumin content from the samples obtained after the tape stripping was performed by liquid chromatography coupled with a mass spectrometry (LC-MS/MS) technique, as already described by Nikolić et al. [20]. Briefly, liquid chromatographic system with Accela autosampler and the pump (Thermo Fisher Scientific, San Jose, CA, USA) was applied. Xterra^®^ MS C18 column (3.5 µm 2.1 × 150 mm; Waters Corporation, Milford, MA, USA) was used for the separation, at 25 °C. A mobile phase consisting of acetonitrile and 0.1% formic acid aqueous solution (70:30 *v*/*v)* was used for isocratic elution, at flow rate of 0.3 mL/min, and run time of 5 min. Mass analyses were conducted on TSQ Quantum Access MAX triple quadripole spectrometer equipped with electrospray ionisation (ESI) source and high-purity nitrogen as nebulising gas. Selected reaction monitoring (SRM) for data collecting was in positive mode, and ESI source and mass spectrometry parameters were at following values: spray voltage—5000 V; vaporizer temperature—400 °C; sheath gas pressure—30 units; ion sweep gas pressure—0 units; auxiliary gas—15 units; ion transfer capillary temperature—250 °C; collision energy—22 V; *m*/*z* 369.2→177.0; capillary offset—35 units; skimmer offset—0 units; peak width—0.7; scan time—200 ms. The data were processed through Xcalibur software v. 2.1.0.1139 (Thermo Fisher Scientific, Waltham, MA, USA).

#### 2.2.10. Statistical Analysis

Except for in vivo studies, which involved the participation of a larger number of subjects, all other measurements were performed through at least three replicates, and the results were presented as mean ± standard deviation. Statistical analysis was performed using OriginPro 8 Data Analysis and Graphing Software (OriginLab Corporation, Northampton, MA, USA). After checking whether the data followed the normal distribution, the analysis was performed through Student’s *t*-test (for two data groups) or analysis of variance (ANOVA) followed by post hoc analysis using the Tuckey HSD test (for more than two data groups). Alternatively, in the cases where the data did not follow normal distribution, the analysis was performed using the Mann–Whitney U test (for two data groups) or using the Kruskal Wallis test (for more than two data groups). Differences with a *p*-value less than 0.05 were considered statistically significant.

## 3. Results and Discussion

### 3.1. In Vitro Characterization of the Selected Nanoemulsions

**Figure 1:**
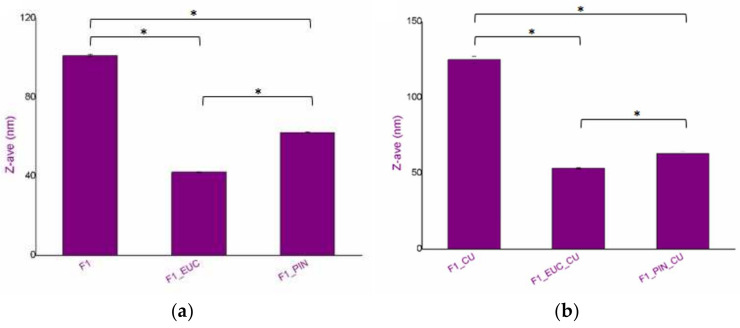
(**a**) Z-ave values for the blank nanoemulsions, (**b**) Z-ave values curcumin-loaded nanoemulsions. * *p* < 0.05 (Kruskal–Wallis ANOVA).

Based on the already conducted and published research work [11,16], the F1 formulation (Table 1) was selected as the starting formulation (nanoemulsion without penetration enhancers). Relying on the previously determined solubilization capacity of the nanoemulsions for curcumin [16], corresponding curcumin-loaded nanoemulsion contained 3 mg/mL of this active ingredients (F1_CU). Essential physical properties of the nanoemulsion are given in the Table 2 and Figure 1.

It could be commented that pH values are appropriate with respect to curcumin’s stability (it undergoes chemical degradation in alkaline conditions) [29] and the anticipated administration route (dermal application). The zeta potential indicated a negative surface charge of the dispersed droplets, but also highlighted good kinetic stability [30]. Curcumin solubilization caused an increase in Z-ave values, which has also been expected due to the finding that pointed out its interfacial localization in this type of the nanoformulation [11].

In terms of skin delivery, nanoemulsions may contribute to better availability of the active ingredient thanks to their specific properties: better substantivity; efficient integration of nanodroplets with corneocytes and lamellarly organized lipids of the stratum corneum, and higher solubilization capacity [4,6]. The selected nanoemulsion was prepared via low-energy nanoemulsification process, thus requiring higher surfactant concentration that would provide appropriate physical stability of the formulation. Surfactants are also known to enhance skin penetration—predominantly through solubilization of superficial skin lipids and protein denaturation [31]. Even though it is favourable from the perspective of dermal availability, safety aspects should also be examined. Prior to any in vivo assessment, tolerability of the selected formulation was evaluated in vitro, through cytotoxicity assay, using normal human keratinocytes (HaCaT cell line)—dominant structural epidermal cells.

**Figure 2:**
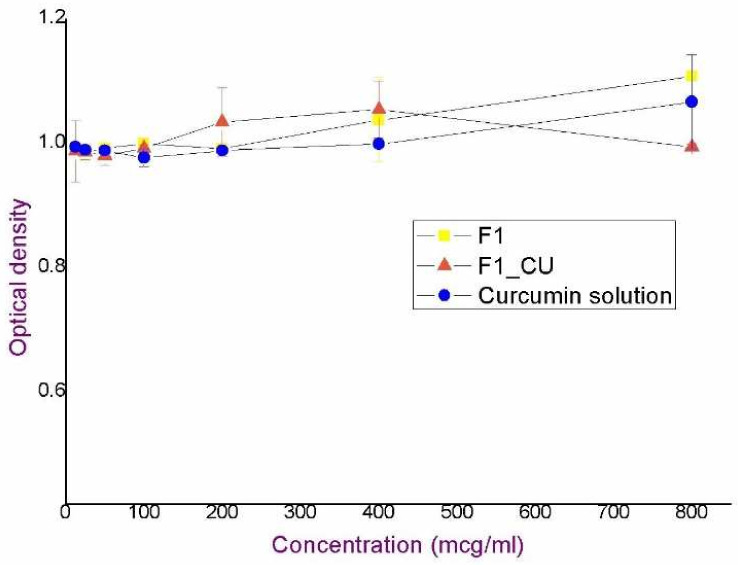
Optical density of normal human keratinocytes treated with curcumin solution, nanoemulsion F1_CU and blank nanoemulsion F1.

By monitoring cell viability, it was shown that there was no significant decrease in the number of metabolically active keratinocytes in the concentration range from 12.5 to 800 μg/mL of curcumin, corresponding dilutions of the nanoemulsion F1_CU and the blank formulation F1, as depicted in the Figure 2. In addition, in the control group of cells, there were no changes in metabolic activity or morphological properties. Obtained findings indicted excellent safety profile of the starting formulation.

Further on, having in mind monoterpenes as penetration enhancers, the F1 formulation was modified so that a part of the MCT was replaced by monoterpene—eucalyptol or pinene, rendering new nanoformulations containing penetration enhancers (Table 1). Thus, the use of chemical penetration enhancers in a complex system (such as a nanoemulsion) could potentially exert even more significant effects in terms of efficiency in dermal delivery [7]. Comparing physical properties of the monoterpene-containing nanoemulsions to the F1, a significant decrease in Z-ave could be explained by their co-stabilizing effect, influencing the curvature of the interface of low-energy nanoemulsions, which has already been elaborated in detail [11]. Interestingly, unlike the previous case, curcumin solubilization did not cause an increase in droplet size in monoterpene-containing formulations. Such a difference in droplet size could also have an impact on skin delivery due to higher contact surface and better adherence to membranes, resulting in a more controlled transport of bioactive compounds into/through the skin [32].

In terms of in vitro safety assessment, formulations with monoterpenes were not evaluated through the MTT assay, as monoterpenes are already known for their cytotoxic effect. Their performances as skin penetration enhancers are predominantly based on the changes in the structure and organization of the lipoprotein matrix of the *stratum corneum*, which can often be associated with adverse skin reactions [6]. This interaction with lipids and proteins is not selective, and a certain degree of “toxicity” of these molecules can be expected in in vitro tests. However, these findings do not prove their general inadmissibility, while results of the in vitro test should not be taken strictly, as experimental conditions do not fully reflect application circumstances.

In addition, application of dermal preparations, in general, is linked to the changes in the barrier properties of the skin, which can cause certain side effects, especially if the formulations contain relatively high proportion of surfactants (as is the case with nanoemulsions prepared by low-energy processes), and some other penetration enhancers. Therefore, in further experimental segments, as a more relevant manner of safety evaluation, presented blank formulations were evaluated in vivo, assessing their irritation potential, while mimicking real application.

### 3.2. In Vivo Safety Study of the Blank Nanoemulsions

**Figure 3:**
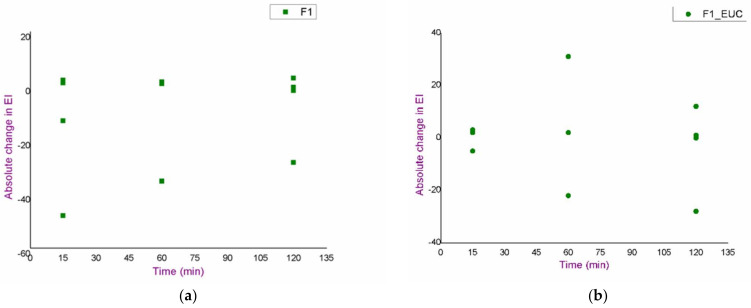

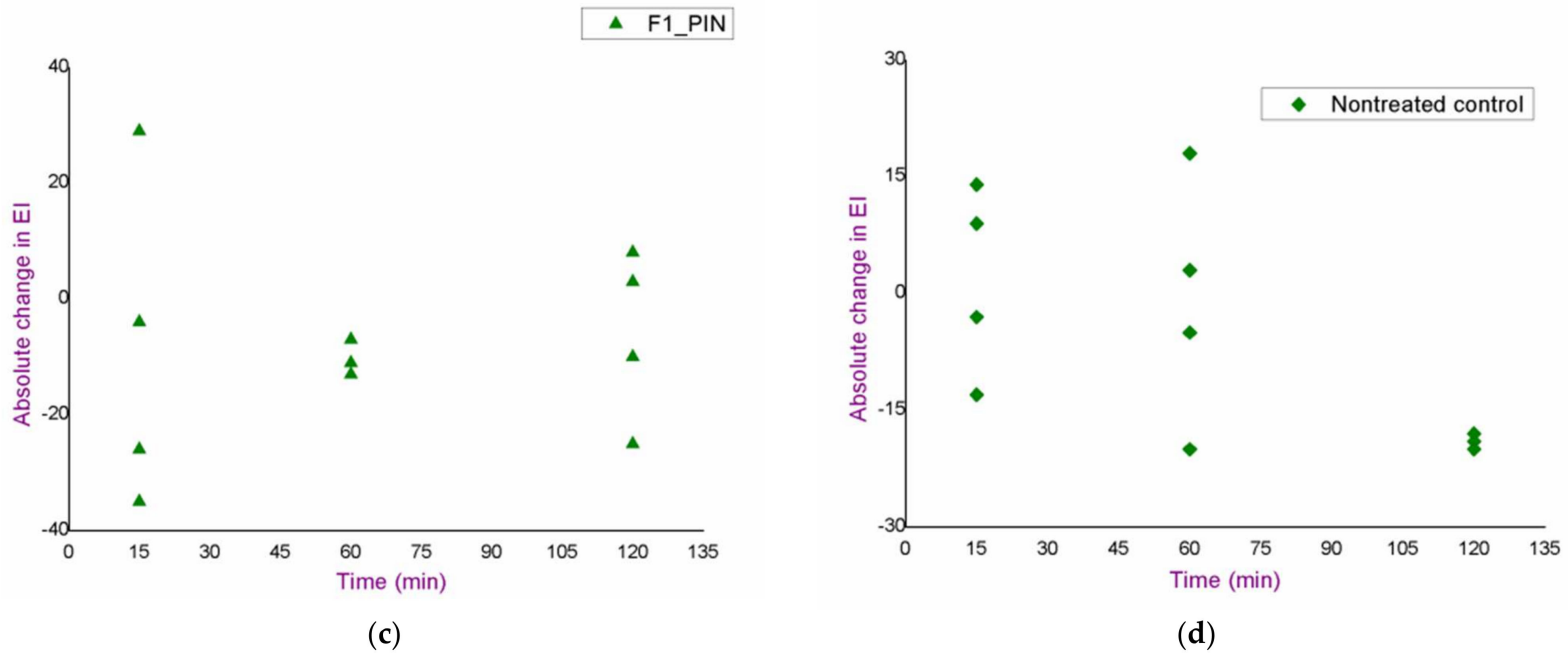
Results of the in vivo safety assessment of the blank nanoemulsions—absolute change in the values of erythema index: (**a**) test place for the formulation F1; (**b**) test place for the formulation F1_EUC; (**c**) test place for the formulation F1_PIN; (**d**) nontreated control.

**Figure 4:**
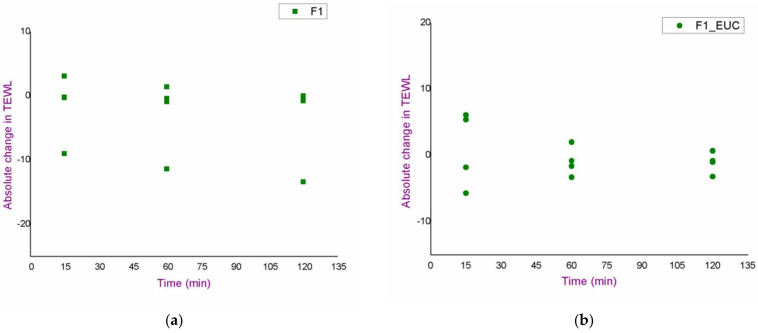

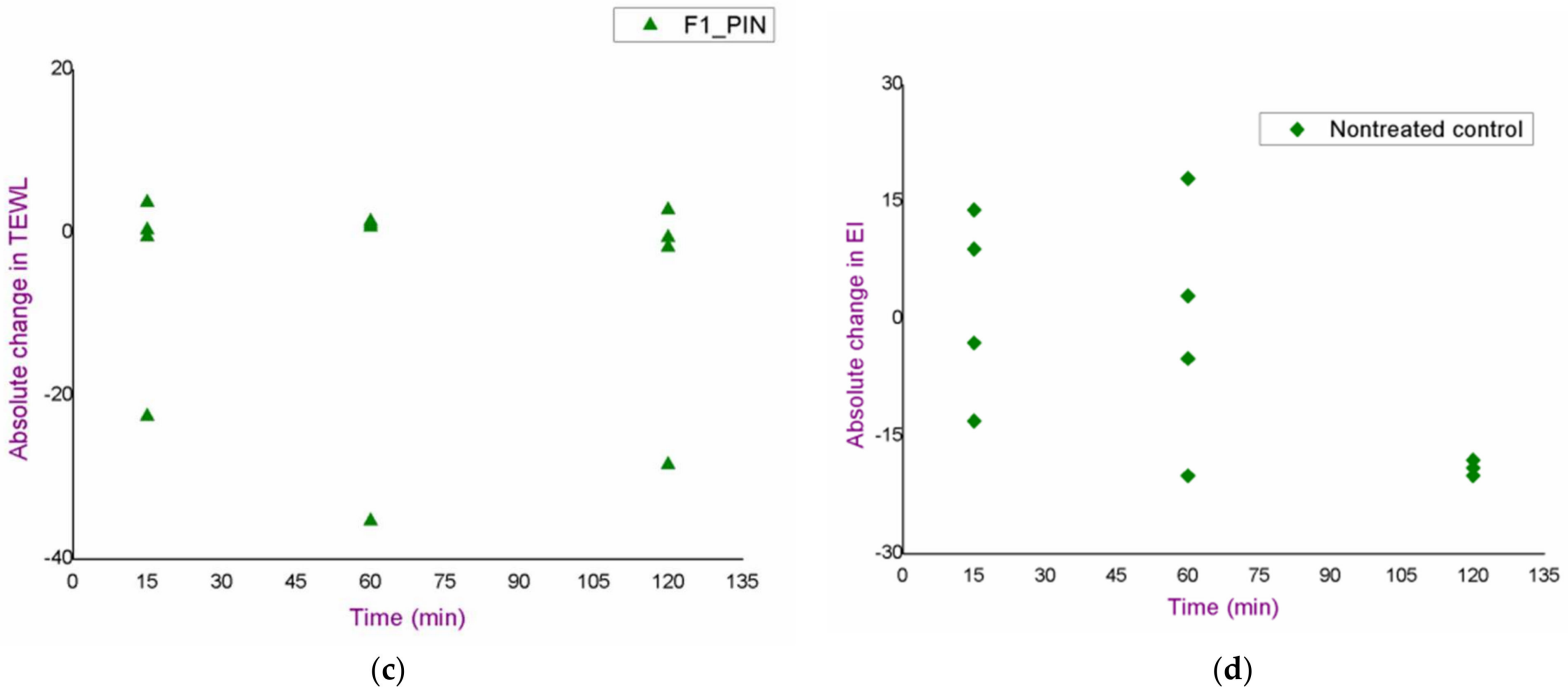
Results of the in vivo safety assessment of the blank nanoemulsions—absolute change in the values of trans-epidermal water loss: (**a**) test place for the formulation F1; (**b**) test place for the formulation F1_EUC; (**c**) test place for the formulation F1_PIN; (**d**) nontreated control.

**Figure 5:**
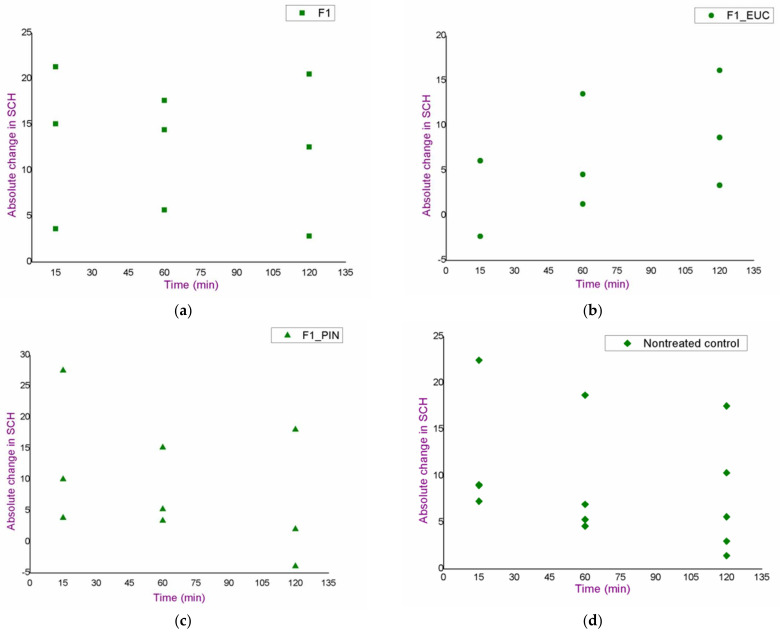
Results of the in vivo safety assessment of the blank nanoemulsions—absolute change in the values of stratum corneum hydration: (**a**) test place for the formulation F1; (**b**) test place for the formulation F1_EUC; (**c**) test place for the formulation F1_PIN; (**d**) nontreated control.

An in vivo safety study of the blank formulations (F1, F1_EUC, F1_PIN) was performed using skin bioengineering techniques, under conditions that more realistically reflect the potential application, by monitoring TEWL, EI and SCH parameters. Aiming to assess the possible dynamics of the changes in the values of skin biophysical parameters, after measuring the basal values, the same parameters were determined 15, 60 and 120 min after application of the samples. Moreover, in order to gain a more complete insight, test sites were observed visually, and volunteers were asked to report any sensations they felt (e.g., tingling, skin tightening, itching…), which might be related to the application of the formulations. Figure 3, Figure 4 and Figure 5. depict absolute changes in the values of the examined parameters at all time points and for each examinee.

As a primary parameter that can quantify changes such as redness and irritation, EI values were measured (Figure 3). Although a trend of increase in EI in relation to the basal value could be observed in some subjects, the given changes could not be characterised as significant given the large inter- and intra-individual variability in this type of measurement. Additionally, in the same subjects, similar changes were observed in the place marked as nontreated control, which further confirmed the conclusion. Furthermore, after visual examination of the treated areas, skin redness or any other visual sign of skin irritation was not noticed, and the volunteers confirmed that the formulations did not cause burning, or any other type of discomfort that would be a consequence of side effects on the skin.

The integrity of the skin barrier itself can be better evaluated by TEWL values, which were uniform during the study, and no increases were observed either relative to basal values, or relative to the nontreated control, for any sample, or at any time point (Figure 4). Such findings indicated a good safety profile, despite the presence of monoterpenes in some of the tested samples.

Changes in SCH values at the F1 formulation test site followed the changes of the same parameter at the nontreated control site. In addition, for the same sample, the increase in SCH was the largest at the first time point (15 min after application of the formulation), but without statistical significance compared to the next two time points (Figure 5), which could be associated with the effect of high water concentration in the formulation. However, changes in SCH at the test sites for F1_EUC and F1_PIN were not significantly different from the basal values, although it was quite reasonable to expect mild skin dehydration at test sites treated with samples containing monoterpenes (due to their volatility).

Due to the relatively small number of participants, a complete and definite conclusion cannot be drawn based on the presented study. However, preliminary results indicated that blank nanoemulsions showed a good safety profile, with no adverse skin reactions.

### 3.3. Selection of Experimental Conditions for the Iontophoresis: An In Vivo Safety Study

An iontophoretic device consists of a source of electrical energy, a microprocessor and electrodes (cathode and anode), which should be in contact with the skin. Depending on the characteristics of the active ingredient, the formulation is positioned below one of the electrodes, labelled as the “active” one, while the circuit is closed by means of another, “return” electrode. In the case of charged active ingredients, the active electrode is the one with the same charge as the active molecule. When the flow of electricity is provided, the active ingredient will move under the influence of the electric field towards the oppositely charged electrode, which is placed at a short distance from the active electrode. On the other hand, the driving force for electroneutral actives is provided by electroosmosis, provided by the convective solvent flow [12,15]. Therefore, in this case, the active electrode is the anode.

With a view to gaining an insight into the effects of iontophoresis on the skin and its barrier properties, in an in vivo study involving eight subjects, EI, TEWL and SCH were again monitored. To cover several different protocols for the application of iontophoresis, three combinations were examined, which included the following conditions:

- continuous current flow for 15 min—15-0;
- discontinuous flow—5 min of flow with 1 min break (3 cycles)—5-1;
- discontinuous flow—3 min flow with 2 min break (5 cycles)—3-2.

Thus, the total duration of current flow through the skin was the same for all examined protocols—15 min.

As the power source, coupled 32 V and 9 V batteries or only a 9 V battery were used. In the first case, when both power sources were used in conjunction, immediately after closing the circuit, a pronounced “burning” sensation and heat would be felt at the place where the electrodes were attached to the skin, which would lead to the skin redness. Due to the unpleasant accompanying feeling, and the potential skin damage that could occur in certain subjects, only the 9 V power source was further used in the experimental setup.

**Figure 6:**
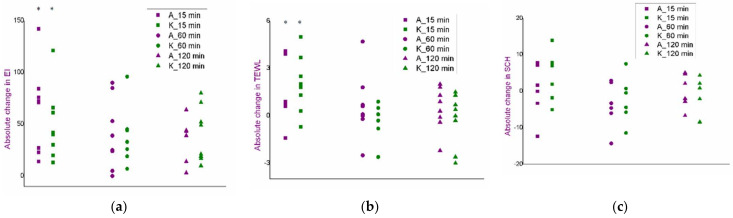
In vivo safety evaluation of the iontophoretic protocol 3-2: absolute changes in the values of erythema index: (**a**), trans-epidermal water loss; (**b**), and stratum corneum hydration; (**c**) at the anode (A) and cathode (K) sites 15, 60 and 120 min after the iontophoresis; ANOVA: * *p* < 0.05 compared to the nontreated control.

**Figure 7:**
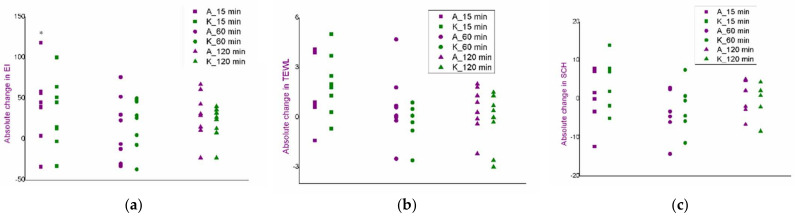
In vivo safety evaluation of the iontophoretic protocol 5-1: absolute changes in the values of erythema index: (**a**), trans-epidermal water loss; (**b**), and stratum corneum hydration; (**c**) at the anode (A) and cathode (K) sites 15, 60 and 120 min after the iontophoresis; ANOVA: * *p* < 0.05 compared to the nontreated control.

**Figure 8:**
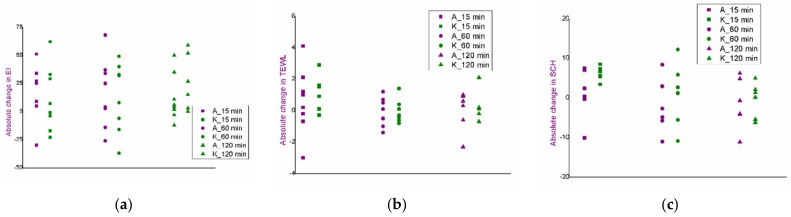
In vivo safety evaluation of the iontophoretic protocol 15-0: absolute changes in the values of erythema index: (**a**), trans-epidermal water loss; (**b**), and stratum corneum hydration; (**c**) at the anode (A) and cathode (K) sites 15, 60 and 120 min after the iontophoresis.

Measurement of EI, TEWL and SCH parameters was performed at sites corresponding to both cathodes and anodes and presented as the absolute changes of the parameter values (Figure 6, Figure 7 and Figure 8), as well as at the site marked as nontreated control.

In the protocol 3-2, after measuring EI and comparing with nontreated control, the values significantly changed at the cathode site and at the anode site, at the first time point. Afterwards, the values of this parameter returned to the basal ones (Figure 6a). Upon removal of the electrodes, slight redness could be seen, which faded away after about 15 min. Additionally, the volunteers themselves stated that with this discontinuous current, they had felt something that they described as “mild and occasional tickling”, which they did not characterise as unpleasant. To additionally examine changes in the barrier properties of the skin, the TEWL values were further analysed. These measurements indicated significant changes at the first time point—both at the cathode site and at the anode site (Figure 6b). In contrast, the SCH values did not show significant changes during the study (Figure 6c).

In the protocol 5-1, the only significant change was an increase in the EI value at the anode site, relative to the nontreated control, and only at the first time point (Figure 7a), but this was not accompanied by a visible increase in redness. All other parameters were uniform during the experiment, with no significant changes compared to the nontreated control (Figure 7b,c).

The protocol with continuous current did not show significant changes in the tested parameters at any test site or at any time point (Figure 8a–c).

**Figure 9:**
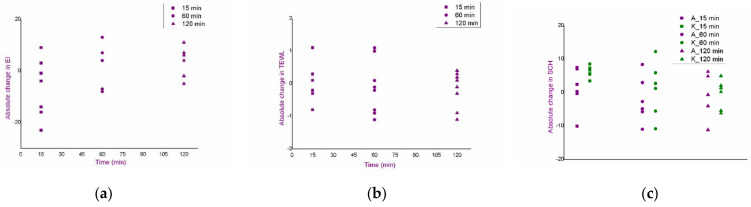
In vivo safety evaluation of the iontophoretic protocol: absolute changes in the values of erythema index: (**a**), trans-epidermal water loss; (**b**), and stratum corneum hydration; (**c**) of the nontreated control site.

Corresponding results of the EI, TEWL and SCH measurements at the nontreated control site are presented in the Figure 9.

Based on the overall analysis, it can be concluded that the examined iontophoresis protocols did not cause undesired changes to the skin. Although in protocols 3-2 and 5-1, increases in EI and/or TEWL values were observed at the first time point, these changes were not of such a nature as to imply a safety risk for the subjects. Furthermore, a certain degree of increase in the values of presented parameters was expected, given the role of iontophoresis in the delivery of active molecules to the skin. For these reasons, all three tested protocols were included in further studies, to evaluate their efficacy in dermal delivery of curcumin.

### 3.4. In Vivo Assessment of Curcumin Penetration through the Superficial Skin Layers after the Application of Curcumin-Loaded Nanoemulsions: Comparison of the Effect of Chemical Penetration Enhancers and Iontophoresis

To assess the penetration of curcumin through the skin after the application of nanoemulsions, as well as after the proposed iontophoresis protocols, a dermatopharmacokinetic analysis was performed using the tape stripping technique, in an in vivo study involving four subjects. When assessing the effect of iontophoresis, only nanoemulsion F1_CU (without chemical penetration enhancers) was used. In this way, it was possible to evaluate the effect of nanoemulsions among themselves (F1_CU, F1_EUC_CU and F1_PIN_CU) regarding the chemical penetration enhancers, and then the effect of iontophoresis by monitoring the penetration of curcumin from the formulation F1_CU independently, and then with the application of all three iontophoresis protocols. In addition, such an experimental setting would indicate the existence of differences in the penetration of curcumin using the iontophoresis with continuous and intermittent current.

The transport of the actives by iontophoresis can be explained through the joint action of 3 mechanisms [15,33]:

- electromigration;
- electroosmosis;
- passive diffusion.

In an electric field, cations move from the anode to the deeper layers of the skin. Analogously, the anions move in the opposite direction, from the cathode. This regulated movement of ions from the active electrode to the deeper layers of the skin is called electromigration—the main electro-transport mechanism responsible for improving the penetration of the drug that is charged under given conditions of application.

It is known that the skin has an isoelectric point (pI) 4–4.5, which, under physiological conditions, provides negative charge. Such an environment favours cation transport, and it can be said that the skin acts as a cation-selective membrane. This selective permeability induces an electroosmotic flow of the solvent in the direction from the anode to the cathode, carrying electroneutral ingredients. Precisely, this type of movement under the influence of electricity represents another electro-transport mechanism—electro-osmosis.

Passive diffusion, as the third transport mechanism, occurs independently of the applied electricity. Due to the hydrophobic nature of the *stratum corneum*, it is negligible for polar and charged molecules, but not without significance for nonpolar molecules.

The relative contribution of each of these transport mechanisms to the total amount of the drug that penetrated primarily depends on the physicochemical characteristics of the active ingredient and on the applied formulation [15].

Due to the negative charge of the skin [15,33], it would be expected that positively charged nanodroplets would interact more efficiently with the skin [34,35]. However, the zeta potential of the nanodroplets of the F1_CU formulation was around −30 mV (Table 2), revealing negative surface charge. Curcumin is characterized by 3 pKa values: pK_a1_ = 7.8, pK_a2_ = 8.5 and pK_a3_ = 9.0 [36], which indicates that it is uncharged at pH values in the range 3–7. Therefore, anode transport was performed (the anode was the “active electrode”), taking the advantage of the electroosmosis as the main transport mechanism. In addition, due to the lipophilicity of curcumin, a certain contribution of passive diffusion is also expected.

**Figure 10:**
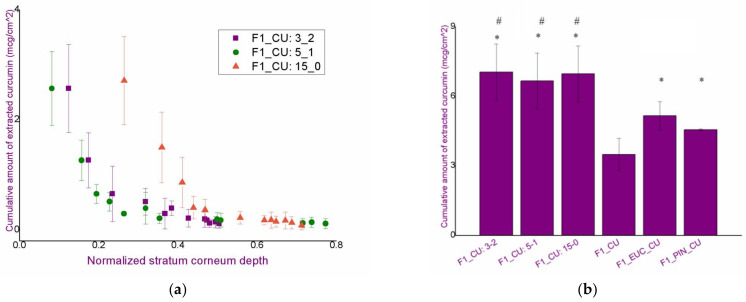
(**a**) Penetration profile of curcumin from the nanoemulsion F1_CU in combination with iontophoresis in three proposed protocols; (**b**) Cumulative amount of curcumin that penetrated through the superficial skin layers (per unit area) from the nanoemulsions F1_CU, F1_EUC_CU and F1_PIN_CU (tape stripping with protocol modification) and from the nanoemulsion F1_CU combined with iontophoresis in three proposed protocols; ANOVA: * *p* < 0.05 compared to F1_CU; # *p* < 0.05 compared to F1_CU, F1_EUC_CU and F1_PIN_CU.

Figure 10a depicts the penetration profile of curcumin from the nanoemulsion F1_CU after applying all three proposed iontophoresis protocols. In all three cases, iontophoresis led to an almost twofold increase in the amount of penetrated active compound per unit area compared to F1_CU without the application of iontophoresis, most probably due to the synergistic effect. Similar was observed by another research group, dealing with zinc phthalocyanine formulations [37]. However, despite literature claims that intermittent current may be more efficient in dermal delivery compared to the continuous one [15], such results were not shown here. Namely, for all three tested modes of exposure, no statistically significant differences were observed in the total amount of curcumin that penetrated through the skin (Q_3-2_ = 7.04 ± 3.21 μg/cm^2^; Q_5-1_ = 6.66 ± 2.11 μg/cm^2^; Q_15-0_ = 6.96 ± 3.21 μg/cm^2^; Q_without iontophoresis (with protocol modification)_ = 3.49 ± 0.70 μg/cm^2^). Additionally, based on the results of the iontophoresis irritation study, a slight advantage of the intermittent protocol could have been expected (primarily 3-2), given the increased EI and TEWL values obtained at the first time points.

It should be noted that the adhesive carrier of the electrode, after removing the electrode from the skin, removes a certain part of the stratum corneum, and thus a part of the curcumin from the surface. To adequately compare the amounts of active ingredient that penetrated the skin with the application of iontophoresis to the amount that penetrated without the application of this method, the protocol was adjusted. Tape stripping on the place where only the curcumin-loaded nanoemulsion was applied (without iontophoresis) was done by placing the adhesive tape with the electrode on that test place, and then removing it after 15 min. In this manner, the protocol was adjusted, so that from each of the examined places, the same amount of curcumin (and the same amount of *stratum corneum* “layers”) was removed, which ensured an appropriate comparison of the results.

The stated lack of difference in the efficiency of continuous iontophoresis delivery in relation to the discontinuous protocol may be a consequence of the relatively small number of subjects in the study, but also of the fact that, despite the discontinuity in the current, the effective flow and pause duration were of the same order of magnitude. In some future experiments, it would be useful to examine the effects obtained at much shorter flow and pause intervals.

In contrast to the findings obtained after the iontophoresis study, by comparing the results after the application of nanoemulsions (without iontophoresis), a significant difference was observed in the total amount of curcumin that penetrated through the superficial skin layers. The most successful formulation was F1_EUC_CU, followed by F1_CU, while the least successful formulation was F1_PIN_CU (Table 3).

The presented finding is not entirely expected, given that the pinene-containing formulation exhibited far less successful delivery of curcumin compared to a nanoemulsion that did not contain monoterpenes. The higher total amount of penetrated curcumin from the formulations F1_EUC_CU and F1_CU compared to F1_PIN_CU could be explained by the differences in the solubility of curcumin in the two applied monoterpenes and in MCT—the highest solubility is in eucalyptole, while pinene is the least efficient solvent [12]. Such differences could lead to higher thermodynamic activity of curcumin after evaporation of monoterpenes from the skin, with consequent better penetration through the *stratum corneum*. On the other hand, better solubility in MCT compared to pinene could lead to a more significant interaction of the active ingredient with endogenous lipids, and consequently more efficient penetration, which could not be achieved after the application of F1_PIN_CU. Thus, although one would expect the monoterpene-containing formulation always to be superior in the context of dermal availability to that without penetration enhancers, the physicochemical properties of the active ingredient and its solubility in selected oil phase components of the nanoemulsion have been shown to be critical. Such a conclusion is supported by already reported results related to in vitro release of curcumin from developed formulation [11]. Even though monoterpene-containing nanoemulsions exhibited higher release rate and total amount of released curcumin, in vivo performance in terms of skin delivery indicated different order of the formulations.

Finally, if the total amounts of penetrated curcumin (per unit area) are compared among iontophoresis and monoterpene-containing nanoemulsions, iontophoresis was significantly more successful in curcumin delivery compared to the formulations with chemical penetration enhancers (Figure 10b). However, as already explained, with the aim of comparing results adequately, a small change in the protocol was performed, using an adhesive patch even at those places where no current was applied. Therefore, obtained numerical values should be only for the aim of relative comparison, and not taken strictly.

Such findings are very significant bearing in mind that curcumin served as a model active ingredient, although it, due to its physicochemical properties, does not represent a first-choice molecule in terms of the application of iontophoresis in dermal delivery. In addition, a certain disadvantage of this study is reflected in the fact that iontophoresis was applied for a relatively short time (15 min). It would be logical to expect larger amounts of curcumin in deeper layers of the skin after applying iontophoresis over a longer period of exposure. However, having in mind the available methodology, in the case of penetration into deeper skin layers, the given quantities could not be adequately quantified. Moreover, the presented absolute values of the total amount of curcumin that penetrated through the superficial layers of the skin in the experiment with iontophoresis are lower than the real ones, considering the already mentioned shortcoming reflected in the removal of a significant part of corneocytes by the adhesive electrode carrier. However, the presented experimental approach provided a valuable insight into the effects and trends that could be expected after the application of electrical current as a type of stimulated dermal delivery of the active ingredient.

## 4. Conclusions

Through the presented experiments it was proven that iontophoresis, under the proposed conditions, in combination with an advanced carrier with nanostructure, provided more efficient dermal delivery of curcumin than the formulations containing chemical penetration enhancers. Additionally, iontophoresis offers several possibilities for further research, with variations in term of applied voltage and exposure time to the current. It would be logical to expect that with the extension of the iontophoresis duration, larger amounts of the active ingredient in the skin could be expected. However, the available methodology for the analysis of dermal availability (tape stripping) could not adequately quantify the amount of the ingredient in the deeper layers of the epidermis, so, in this work, only 15-min exposure was examined.

Finally, improvement of an efficient mobile adhesive system for iontophoresis would be a practical contribution in the field of dermal drug application.

## Figures and Tables

**Table 1:** Qualitative and quantitative (%) composition of the blank and curcumin-loaded formulations (with or without chemical penetration enhancers).

Formulation	MCT	Eucalyptol	Pinene	Curcumin	Soybean Lecithin	Polysorbate 80	Water
F1	10	/	/	/	1	9	80
F1_EUC	5	5	/				
F1_PIN	5	/	5				
F1_CU	10	/	/	0.3			79.7
F1_EUC_CU	5	5	/				
F1_PIN_CU	5	/	5				

**Table 2:** pH and zeta potential values of the blank and corresponding curcumin-loaded nanoemulsions.

Formulation	pH	Zeta Potential (mV)
F1	4.78 ± 0.04	−33.07 ± 0.95
F1_EUC	6.25 ± 0.03	−21.17 ± 1.17
F1_PIN	4.65 ± 0.01	−39.50 ± 1.30
F1_CU	5.36 ± 0.03	−30.33 ± 1.81
F1_EUC_CU	6.23 ± 0.01	−31.10 ± 0.10
F1_PIN_CU	5.80 ± 0.03	−22.27 ± 0.06

**Table 3:** Cumulative amount of curcumin (from the tested nanoemulsions) that penetrated through the superficial skin layers per unit area.

Formulation	Cumulative Amount of Curcumin That Penetrated through the Stratum Corneum per Unit Area (μg/cm^2^)
F1_CU	30.62 ± 2.62
F1_EUC_CU	34.24 ± 5.68 ^#^
F1_PIN_CU	21.61 ± 4.01 *

ANOVA: * *p* < 0.05 compared to F1_CU and F1_EUC_CU; ^#^ *p* < 0.05 compared to F1_CU.

## Data Availability

The data presented in this study are available on request from the corresponding author and will be available in the repository of the University of Belgrade—Faculty of Pharmacy: https://farfar.pharmacy.bg.ac.rs/.

## Supplementary Materials

The following supporting information can be downloaded at https://www.mdpi.com/article/10.3390/pharmaceutics14061144/s1. Stability study of the designed nanoemulsions: Figure S1: Selected parameters of physical stability of nanoemulsions F1 and F1_ CU; Figure S2: Selected parameters of physical stability of nanoemulsions F_EUC and F1_EUC_CU; Figure S3: Selected parameters of physical stability of the nanoemulsions F1_PIN and F1_PIN_CU; Figure S4: Curcumin content in curcumin-loaded nanoemulsions 24 h after preparation and after one year.
