# Supplementary material for: Chemical vs. Physical Methods to Improve Dermal Drug Delivery: A Case Study with Nanoemulsions and Iontophoresis
Source: Pharmaceutics. 2022 May 27;14(6):1144. doi: 10.3390/pharmaceutics14061144 (PMC9229366; doi:10.3390/pharmaceutics14061144)
Supplement: Supplementary file 1 [file pharmaceutics-14-01144-s001.zip › pharmaceutics-1719166-supplementary.pdf]

Supplementary material section

Chemical vs. Physical Methods to Improve Dermal Drug Delivery: A Case Study with Nanoemulsions and Iontophoresis

Ines Nikolić [1], Mitar Simić [2], Ivana Pantelić [1], Goran Stojanović [2], Jelena Antić Stanković [3], Bojan Marković [4,*] and Snežana Savić [1]

1 Department of Pharmaceutical Technology and Cosmetology, University of Belgrade – Faculty of Pharmacy, 11 000 Belgrade, Serbia; ines.nikolic@pharmacy.bg.ac.rs; ivana.pantelic@pharmacy.bg.ac.rs; snezana.savic@pharmacy.bg.ac.rs
2 Department of Electronics, University of Novi Sad – Faculty of Technical Sciences, 21 000 Novi Sad, Serbia; mitar.simic@uns.ac.rs, sgoran@uns.ac.rs
3 Department of Microbiology and Immunology, University of Belgrade – Faculty of Pharmacy, 11 000 Belgrade, Serbia; jelena.stankovic@pharmacy.bg.ac.rs
4 Department of Pharmaceutical Chemistry, University of Belgrade – Faculty of Pharmacy, 11 000 Belgrade, Serbia; bojan.markovic@pharmacy.bg.ac.rs

*Correspondence: bojan.markovic@pharmacy.bg.ac.rs; Tel.: +381 11 3951 341

Stability study of the designed nanoemulsions

Throughout the storage period, all formulations had uniform rheological properties, and were without signs of phase separation. For curcumin-loaded formulations, the absence of precipitation was confirmed by polarization microscopy.

When it comes to the average hydrodynamic diameter, in the formulation F1, statistically significant increase was observed only compared to the first time point (after 24h), while in the rest of the monitored period the size of the dispersed droplets remained without significant changes (Figure S1). This finding could be related to the consolidation of the system at the initial time points. Similarly, the PDI at all time points was below 0.2, with no statistically significant changes over time, suggesting a relatively narrow size distribution, without coalescence (Figure S1).

On the other hand, terpene-containing nanoemulsions (F1_EUC and F1_PIN) showed significantly lower values of the average hydrodynamic diameter compared to the F1. Statistically significant increase in the value of Z-ave from the first to the second time point was recorded for the F1_EUC, but always remained below 100 nm (Figure S2).

The formulation F1_PIN, in contrast to F1 and F1_EUC, showed a significant change in size, but only after 6 months (Figure S3). Electron paramagnetic resonance spectroscopy showed that pinene was localized in the interfacial layer, interacting with nonpolar tails of surfactants. However, its structure is such that it does not favour the formation of hydrogen bonds, so the interfacial layer does not have this additional type of interaction that could contribute to physical stability, as is the case with F1_EUC. Its presence on the interface potentially reduces the density of the stabilizers, without additional interactions with the water molecules that are close to the interface, probably causing the observed increase in droplet size over time. On the other hand, it is noteworthy to mention that those significant changes in size over time did not occur for the curcumin-loaded nanoemulsion containing pinene (F1_PIN_CU), which may be due to the presence of this molecule at the interface which, despite its hydrophobicity, possesses the ability to form hydrogen bonds with the interfacial water. Thus, it can form slightly oriented water dipoles at the interface, preventing droplet coalescence.

Zeta potential, as a measure of surface charge, had stable values during the entire period, without statistically significant changes. Although the electrostatic mechanism is not the only mechanism responsible for the stabilization

of the given nanoemulsions, experimental data show that nanoemulsions with negatively charged amphiphilic monolayers show significantly better stability compared to formulations with positively charged or electroneutral interfaces. Due to the existence of electrostatic repulsion between opposite sides of the interface, lower packing density is provided.

Monitoring the pH value, it was determined that all the tested formulations remained in the range that favours the chemical stability of curcumin, but also the intended route of application, with a statistically significant decrease after one year only in the formulation F1_EUC_CU. However, the obtained values could not be related to a certain type of formulation instability.

As curcumin is extremely prone to oxidative degradation, the biggest challenge is to preserve its chemical stability in the formulation. During the one-year follow-up of the nanoemulsions, there was no significant decrease in the curcumin content (Figure S4).

Finally, it could be concluded that during the storage period, at room temperature, no signs of physical or chemical or instability of the blank nanoemulsions F1 and F1_EUC were observed, while in the formulation F1_PIN a significant increase in the average droplet size was observed, but only after 6 months. All curcumin-loaded nanoemulsions proved to be stable - both physically and chemically, during this one-year follow-up.

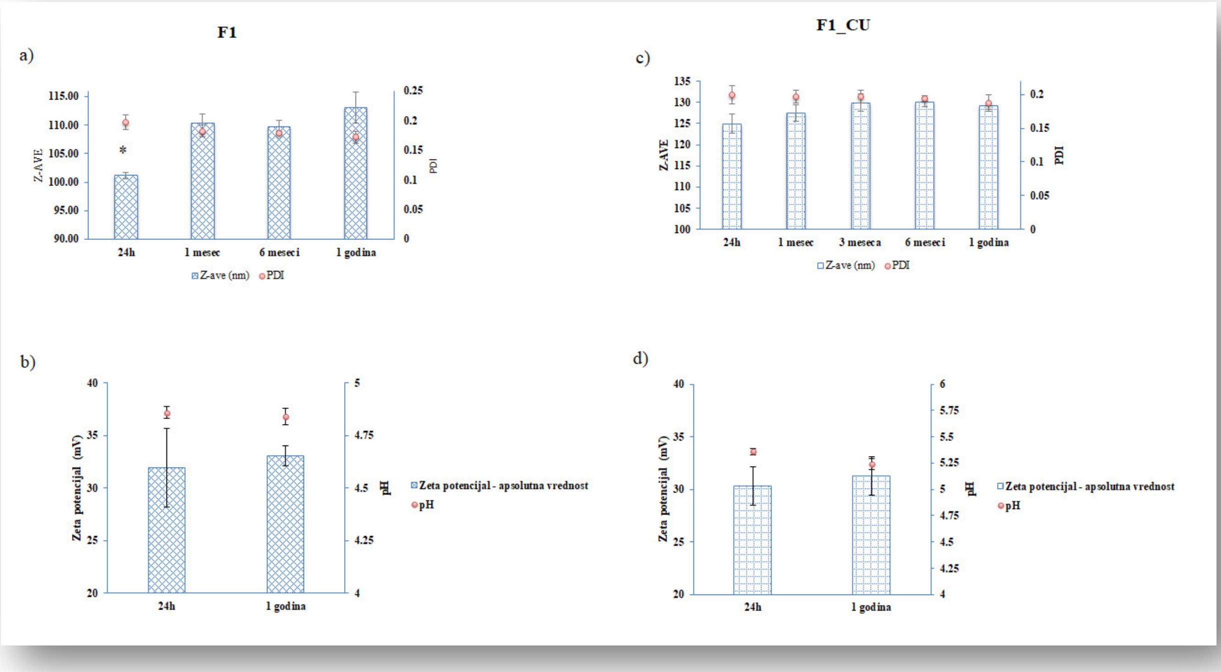

Figure S1. Selected parameters of physical stability of nanoemulsions F1 and F1_ CU

ANOVA: * $p < 0.05$ (Z-ave value of the formulation F1 24 h after production in relation to Z-ave value at all other time points; n = 3, mean ± standard deviation)

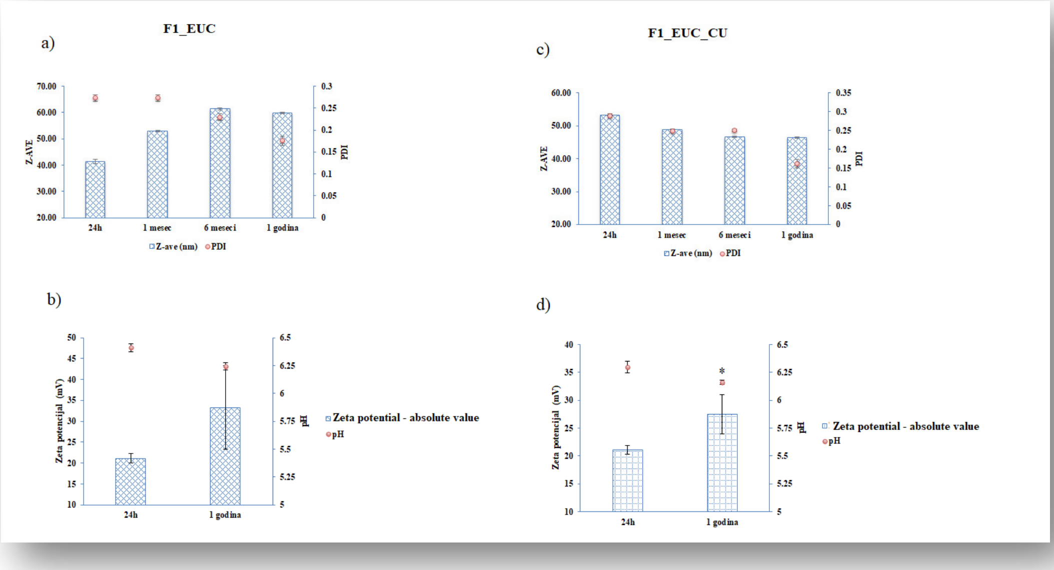

Figure S2. Selected parameters of physical stability of nanoemulsions F_EUC and F1_EUC_CU

Student's t-test: * $p < 0.05$ (pH of the formulation F1_EUC_CU_3 after one year in relation to the initial value; n = 3, mean ± standard deviation)

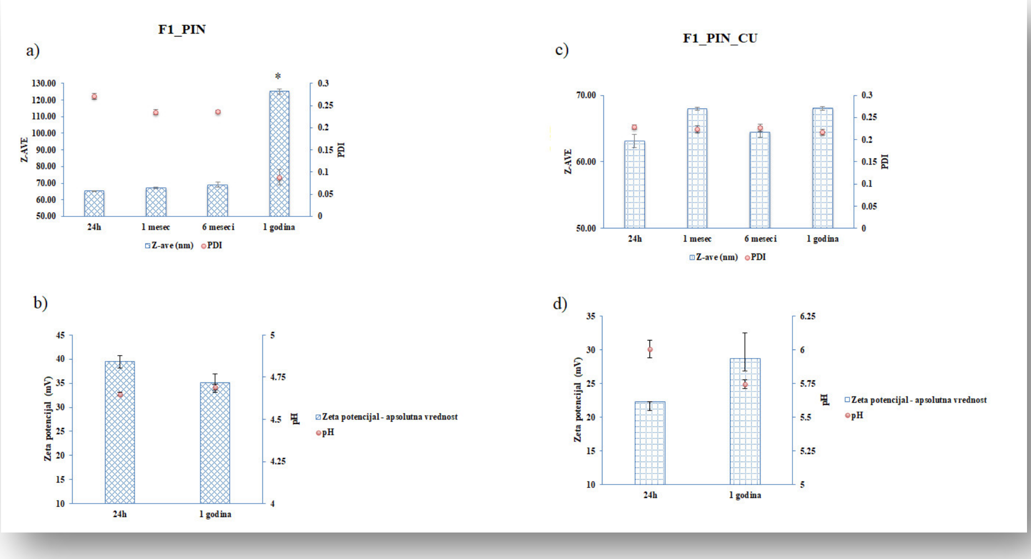

Figure S3. Selected parameters of physical stability of the nanoemulsions F1_PIN and F1_PIN_CU

ANOVA: * $p < 0.05$ (Z-ave formulations F1_PIN after one year in relation to Z-ave at all other time points; n = 3, mean value ± standard deviation)

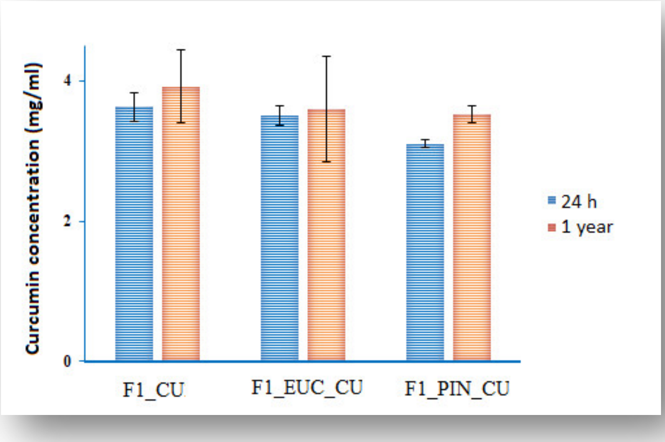

Figure S4. Curcumin content in curcumin-loaded nanoemulsions 24 h after preparation and after one year (n = 3, mean values ± standard deviation)
